# Supplementary material for: Antidiabetic drug utilization pattern, affordability and cost analysis in Iran from 2009 to 2021: A need to revise guideline
Source: PLoS One. 2025 Oct 31;20(10):e0333186. doi: 10.1371/journal.pone.0333186 (PMC12578154; doi:10.1371/journal.pone.0333186)
Supplement: Attachment — Submitted filename: second rebuttal letter.docx [file pone.0333186.s003.docx]

Reviewer 2:

Language and Style

While the study contains valuable data, the manuscript still suffers from grammatical errors, which may hinder reader comprehension. A thorough professional English language edit is strongly recommended.

Thank you for your suggestion, the article was edited and improved in terms of English.

Figures and Tables

Readability: The current use of light ash colour in Figures 1–6 makes key elements difficult to read. Please use black for improved visibility. For example, in Figure 5, the "Minimum daily wage" lines and the horizontal axis years would be more legible if displayed in black.

According the suggestion of reviewer 2 and editor the ash color changed to black in all part of the figures so visibility od them enhanced.

Figure Titles: Please remove titles from inside the figures. For instance, Figure 2 is titled “Antidiabetic drugs expenditure” in the caption but also contains the title “Antidiabetic medicine expenditure” within the figure itself. This duplication detracts from the professional appearance. The same applies to other figures—retain the title only in the caption, not within the figure.

Titles removed from inside the figures.

ACADEMIC EDITOR:

While the manuscript has merit, the language must undergo a thorough professional edit to address grammatical errors, awkward phrasing, and inconsistent tense usage, ensuring clarity and fluency throughout.

In addition, the presentation of figures must be improved by enhancing visibility and removing embedded titles to avoid duplication with captions.

Any changes made must be indicated as tracked changes.

These refinements are essential to enhance readability and professional presentation. Please note that failure to fully address these concerns in the revised submission may result in rejection of the manuscript.

All corrections and improvements to the article's text have been made based on your suggestions.
